# Urinary DNA Methylation and Hydroxymethylation Dynamics as Candidate Biomarkers of Occupational Chemical Exposure—An Exploratory Pilot Study

**DOI:** 10.3390/ijms27146411

**Published:** 2026-07-19

**Authors:** Andrzej R. Reindl, Kamil Pajak, Mateusz Podlasiewski, Alicja M. Debska Slizien, Alicja Trawinska, Tomasz Ksiazek, Agnieszka Siomek-Gorecka, Fabian Lesniewski, Rafal Rozalski

**Affiliations:** 1 Department of Environmental Toxicology, Faculty of Health Sciences, Medical University of Gdansk, 80-210 Gdansk, Polandalicja.trawinska@gumed.edu.pl (A.T.); 2 Department of Nephrology, Transplantology and Internal Medicine, Faculty of Medicine, Medical University of Gdansk, 80-210 Gdansk, Poland; 3 Department of Clinical Biochemistry, Faculty of Pharmacy, Collegium Medicum in Bydgoszcz, Nicolaus Copernicus University in Toruń, 85-950 Bydgoszcz, Poland

**Keywords:** DNA methylation, 5-hydroxymethylcytosine, 8-oxodG, occupational exposure, isocyanates, melamine, urinary biomarkers, epigenetics

## Abstract

DNA methylation (5-mdC) and its oxidative derivatives reflect interactions between environmental exposures and genome regulation, yet their short-term responsiveness to occupational factors remains insufficiently characterized. This exploratory pilot study evaluated whether a standard workweek is associated with measurable changes in urinary epigenetic and oxidative DNA modification profiles across distinct occupational settings. A cohort representing three exposure scenarios—cyanoacrylate-based cosmetic services, toluene diisocyanate (TDI) exposure, and polyurethane (PUR) foam processing—was examined using a paired pre-/post-exposure design. Urinary levels of 5-methyl-2′-deoxycytidine (5-mdC), 5-hydroxymethylcytosine (5-hmCyt), 5-hydroxymethyl-2′-deoxycytidine (5-hmdC), 8-oxo-7,8-dihydro-2′-deoxyguanosine (8-oxodG), 5-hydroxymethyl-2′-deoxyuridine (5-hmdU), and 5-hydroxymethyluracil (5-hmUra) were quantified, and an epigenetic turnover ratio (5-hmdC/5-mdC) was derived. Although none of the observed shifts achieved formal statistical significance after False Discovery Rate (FDR) adjustment (*p*_FDR > 0.05), exposure-specific, directionally consistent non-significant trends were noted across cohorts. The isocyanate-exposed group demonstrated coordinated reductions in 5-mdC and 5-hmdC accompanied by an increase in 5-hmdU. The PUR-processing group exhibited the most pronounced response, including a marked elevation in 5-hmdU and an increased turnover ratio. In contrast, the beauty sector showed minimal median shifts but substantial inter-individual variability. These preliminary findings suggest that urinary epigenetic and oxidative DNA markers might reflect short-term, exposure-related biological trends, warranting further evaluation.

## 1. Introduction

Maintaining the chemical and structural stability of deoxyribonucleic acid (DNA) is a fundamental prerequisite for cellular homeostasis. Modern molecular biology perceives the transition from physiological health to pathological states, including carcinogenesis, as a synergistic result of genetic mutations, epigenetic dysregulation, and oxidative damage [1]. While genetic mutations alter the primary sequence of nitrogenous bases, epigenetic modifications and oxidative lesions modify their chemical properties without changing the template sequence, profoundly impacting gene expression regulation and chromosomal stability [2]. A cornerstone of this epigenetic regulation is the precise pattern of cytosine methylation. The presence of 5-methyl-2′-deoxycytidine (5-mdC) in specific genomic regions, particularly CpG islands, is essential for embryonic development and tissue differentiation [3,4]. This modification, catalyzed by DNA methyltransferases, including DNMT1, DNMT3A, and DNMT3B, affects chromatin structure and gene expression. Methylation of promoter regions is typically associated with transcriptional repression, whereas methylation of repetitive sequences contributes to maintaining genomic stability [5,6].

However, the epigenetic landscape is highly dynamic. The discovery of the Ten-eleven translocation (TET) enzyme family has redefined DNA demethylation as an active, regulated process where 5-mdC is oxidized to 5-hydroxymethyl-2′-deoxycytidine (5-hmdC), often referred to as the “sixth base” of the genome [7,8,9]. Although 5-hmdC serves as a stable epigenetic mark correlated with transcriptional activity and chromatin plasticity, its loss is recognized as an early hallmark of genomic instability and malignant transformation [10,11]. In pathological contexts, such as cancer, global DNA hypomethylation and the reduction of 5-hmdC levels drive chromosomal instability and phenotypic plasticity, reflecting long-term changes in epigenetic regulation rather than direct DNA damage [12,13].

Parallel to epigenetic signaling, environmental and occupational stressors often trigger the overproduction of reactive oxygen species (ROS), including hydroxyl radicals (·OH) and singlet oxygen (^1^O_2_). These species target nitrogenous bases, with guanine being particularly susceptible due to its low ionization potential. The resulting lesion, 8-oxo-7,8-dihydro-2′-deoxyguanosine (8-oxodG), is the gold standard for assessing chronic oxidative stress [14]. Beyond guanine, oxidative attack on thymine or the enzymatic deamination of 5-hmdC leads to the formation of 5-hydroxymethyluracil (5-hmUra) and 5-hydroxymethyl-2′-deoxyuridine (5-hmdU) [15,16]. These modified pyrimidines, although occurring less frequently than purine lesions, can induce replication errors if not excised by specific glycosylases of the base excision repair (BER) pathway, such as TDG or MBD4 [17,18].

While much research traditionally focuses on tissue-specific alterations, urine has emerged as a superior, non-invasive matrix for longitudinal biomonitoring, particularly in occupational settings [19]. The urinary excretion of modified nucleosides reflects the global turnover of DNA damage and the real-time efficiency of the body’s repair systems [20]. Recent clinical breakthroughs have demonstrated that urinary signatures of 5-hmdC and 5-mdC provide a comprehensive view of the body’s epigenetic health, often with higher sensitivity than plasma-based assays [21]. The clinical utility of these markers is underscored by their prognostic value in oncology, where elevated urinary 8-oxodG and 5-hmUra may serve as diagnostic markers in colorectal and lung cancers [22,23,24,25,26]. Furthermore, these markers are increasingly relevant in neurodegenerative research, where 8-oxodG accumulation in both nuclear and mitochondrial DNA is linked to the pathogenesis of Alzheimer’s and Parkinson’s diseases [27,28].

The accurate quantification of these markers requires sophisticated analytical methodologies. High-pressure liquid chromatography with electrochemical detection (HPLC-ECD) remains a staple for 8-oxodG analysis due to its high selectivity [29]. However, liquid chromatography-tandem mass spectrometry (LC-MS/MS and 2D UPLC-MS/MS) is now considered the gold standard, allowing for the simultaneous measurement of epigenetic and oxidative profiles with the use of stable isotope-labeled internal standards [30,31]. While immunoenzymatically assays (ELISA) offer a cost-effective alternative, they frequently overestimate concentrations due to antibody cross-reactivity, making chromatographic methods preferable for high-precision studies [32]. For genome-wide mapping, techniques such as oxidative bisulfite sequencing (oxBS-seq) or the non-destructive TET-assisted pyridine borane sequencing (TAPS) have replaced traditional bisulfite methods to accurately distinguish between 5-mdC and 5-hmdC [33,34]. Furthermore, recent advancements in third-generation technologies, such as nanopore sequencing, now enable the direct, amplification-free detection of both 5-mdC and 5-hmdC on native DNA strands [35].

Occupational exposure to hazardous chemicals remains a major global health challenge. Estimates from the International Labour Organization (ILO) and the World Health Organization (WHO) suggest that over 1 billion workers worldwide are exposed to hazardous substances, leading to approximately 1 million deaths annually due to occupational cancers and related pathologies [36]. In the European Union, data from EU-OSHA indicate that approximately 17% of employees report handling chemical products or substances for at least a quarter of their working time [37]. These figures underscore the urgent need for sensitive, non-invasive “early-warning” biomarkers that can detect biological strain long before clinical symptoms manifest.

In light of these challenges, this study aims to evaluate the dynamic changes in urinary epigenetic markers (5-mdC, 5-hmCyt, 5-hmdC) and oxidative damage products (8-oxodG, 5-hmdU, 5-hmUra) in workers across three distinct sectors. By comparing baseline levels (pre-exposure) with post-exposure samples after a standard workweek, we seek to determine whether these urinary molecular signatures can serve as early-warning biomarkers of occupational genotoxic stress, potentially identifying high-risk individuals long before the onset of clinical pathology.

## 2. Results

### 2.1. Quantitative Analysis of Urinary DNA Modifications Across Occupational Groups

**Figure 1:**
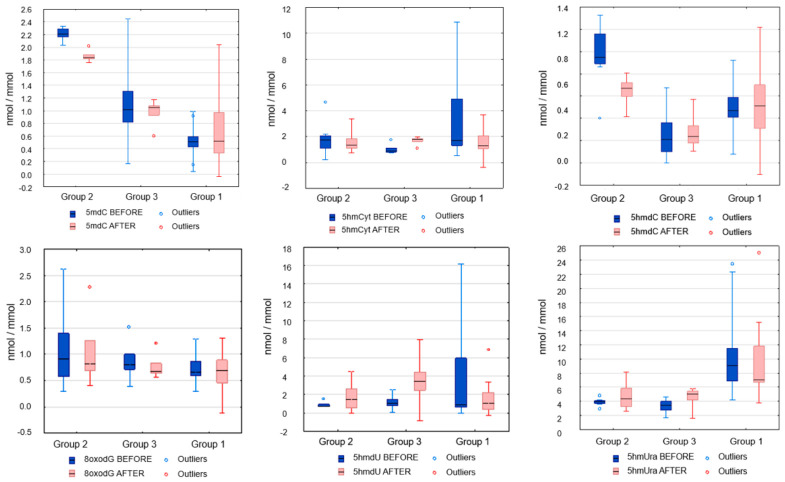
Dynamics of urinary epigenetic and oxidative DNA modifications across three studied groups. Panels represent: 5-mdC (5-methyl-2′-deoxycytidine), 5-hmCyt (5-hydroxymethylcytosine), 5-hmdC (5-hydroxymethyl-2′-deoxycytidine), 8-oxodG (8-oxo-7,8-dihydro-2′-deoxyguanosine), 5-hmdU (5-hydroxymethyl-2′-deoxyuridine), and 5-hmUra (5-hydroxymethyluracil). *X*-axis labels: 1—Beauty sector (lash stylists), 2—Isocyanates exposure, 3—Polyurethane foam processing (melamine/dust). Boxes represent the interquartile range (IQR) with the median indicated by the central line; whiskers represent the range of the data.

The analysis of urinary biomarkers revealed distinct molecular trajectories across the studied cohorts (Figure 1).

Group 2 (Isocyanates) manifested the most distinct epigenetic alterations. A substantial reduction in both the median levels and the interquartile range (IQR) of 5-mdC and 5-hmdC was recorded between the pre- (before) and post-exposure (after) phases, indicating a systemic depletion of DNA methylation markers.

Conversely, the Beauty sector (Group 1) was characterized by inter-individual heterogeneity, particularly regarding baseline 5-hmCyt levels (before), which exhibited a markedly broader distribution compared to other cohorts. Following the workweek, a narrowing of this distribution and a minor median decrease were observed. In contrast, Group 3 (Foam cutting) demonstrated an opposite trend for 5-hmCyt, with a median upward shift recorded between pre- and post-exposure.

While 8-oxodG levels remained relatively stable across the cohorts, pyrimidine-derived oxidative markers demonstrated high sensitivity to specific industrial stressors.

An elevation in 5-hmdU was recorded in Group 3 (Foam cutting), where both the median and IQR for post-exposure samples shifted markedly higher compared to baseline. A parallel, though less pronounced, upward trend in 5-hmdU was observed in Group 2.

Furthermore, 5-hmUra levels increased uniformly in the foam-cutting cohort. In the beauty sector (Group 1), 5-hmUra concentrations remained elevated throughout the workweek, though a slight reduction in variance was noted by the Friday measurement.

### 2.2. Workweek-Induced Shifts

The calculation of median percentage change (%Δ) confirmed highly sector-specific molecular trajectories (Table 1).

In Group 1 (Lash Stylists/Beauty), the median shifts for the primary epigenetic markers were minor, as exemplified by a negligible increase in 5-mdC (+0.7%). However, this cohort was characterized by high inter-individual variance. While the median remained stable, specific hyper-responders within the group demonstrated extreme deviations from the baseline. Concurrently, a noticeable decrease was observed in median 5-hmCyt (−23.2%) and 5-hmUra (−21.8%) levels.

Group 2 (Isocyanates exposure) demonstrated a synchronized depletion of key epigenetic marks. Median levels of 5-mdC and 5-hmdC decreased by 17.2% and 29.1%, respectively, alongside a reduction in 5-hmCyt (−22.8%). In contrast to the decline in epigenetic markers, a profound oxidative shift was observed in this cohort, evidenced by a 103.3% median increase in urinary 5-hmdU.

Group 3 (Polyurethane/PUR processing) exhibited a trend toward a more pronounced biological response across the study. The cohort was characterized by a 221.5% median increase in urinary 5-hmdU, reflecting an upward trend observed across the group. An apparent accumulation was also recorded for markers of epigenetic turnover and active demethylation, with 5-hmCyt increasing by 108.7% and 5-hmdC by 14.1%. Conversely, the median level of 5-mdC showed only a mild increase (+3.75%), while 8-oxodG levels decreased by 14.9%.

### 2.3. Analysis of the Epigenetic Turnover Ratio

To evaluate the systemic efficiency of the DNA demethylation machinery, we calculated the Epigenetic Turnover Ratio, defined as the molar ratio of 5-hmdC to 5-mdC (Ratio = [5-hmdC]/[5-mdC] × 10^−3^). Because 5-hmdC is the direct oxidation product of 5-mdC via the TET enzyme family, we hypothesized that the relative proportion of these two circulating metabolites could serve as a preliminary index of whole-body oxidation flux.

We explicitly acknowledge, however, that this specific equation is proposed de novo in this exploratory study and currently lacks experimental validation. While conceptualized as a theoretical proxy for TET pathway activity, urinary excretion profiles represent a pooled, systemic clearance of nucleosides and do not perfectly reflect localized, tissue-specific intracellular genomic modifications. Therefore, this ratio must be treated strictly as a hypothetical, non-invasive surrogate marker of global biological turnover rather than a validated, direct measurement of cellular demethylation efficiency. The temporal dynamics of this ratio across the workweek are summarized in Table 2.

#### Differential Modulation of the TET-Mediated Pathway

The ratio analysis revealed divergent molecular strategies adopted by the genome in response to different industrial stressors.

Group 2 (Isocyanates) exhibited a synchronized reduction in both the global methyl pool and the turnover ratio (−16.3%). This pattern indicates that isocyanate exposure leads to a stagnant epigenetic landscape, likely caused by the simultaneous depletion of 5-mdC and the enzymatic inhibition of TET-mediated oxidation. Such a state may reflect a compromised capacity for epigenetic reprogramming and maintenance of genomic plasticity.

In contrast, Group 3 (Foam processing) was characterized by a profound 133.3% increase in the turnover ratio. The rise in 5-hmdC relative to 5-mdC suggests activation of the DNA demethylation pathway. This hyper-turnover state suggests that the combination of polymer dust and melamine triggers a systemic stress response, where the genome undergoes rapid enzymatic oxidation as a potential compensatory or repair mechanism against particulate-induced genotoxicity.

In Group 1 (Beauty sector), the ratio remained remarkably stable (+6.5%), suggesting that despite individual variations in absolute biomarker levels, the global equilibrium between DNA methylation and demethylation remains functionally preserved within this occupational microenvironment.

A comprehensive summary of descriptive statistics, Wilcoxon paired sequence test results, and effect size indicators for each occupational exposure group is provided in Table 3. Correction for multiple testing using the Benjamini–Hochberg (FDR) procedure indicated that none of the analyzed changes before and after exposure reached formal statistical significance across the entire analysis (*p*_FDR > 0.05). However, analyses of standardized effect-size indicators (r) and median changes revealed distinct biological profiles between this group and others with different exposure patterns.

In Group 1 (Beauty, *n* = 10–13), exposure did not result in rapid shifts in the genetic profile. Most markers (5mdC, 5hmdC, 8ocodG, 5hmdU, 5hmUra) exhibited no effect or only a small effect size estimates (r range: 0.011–0.204). A moderate effect size was observed for 5hmCyt (median before = 1.70; median after = 1.30; T = 14; *p* = 0.169; r = 0.435); however, this result was not statistically significant.

In Group 2 (Isocyanates, *n* = 4), changes in the dynamics of biological responses were observed. The DNA methylation marker 5mdC exhibited a consistent downward trend, with the median decreasing from 2.21 before exposure to 1.83 after exposure. This corresponded to the lowest attainable *p*-value for this sample size (T = 0; *p* = 0.068) and therefore did not reach statistical significance. A very large effect size was estimated (r = 0.913); however, this should be interpreted with caution due to the extremely small sample size and the risk of effect size inflation. Other markers yielded moderate to large effect size estimates (r = 0.535–0.548), but none were statistically significant.

A similar pattern was observed in Group 3 (PUR processing, *n* = 4), with repeated increases in the studied biomarkers. For 5hmUra, the median increased from 3.43 to 5.04 (T = 0; *p* = 0.068), corresponding to a large effect size (r = 0.913), which should again be interpreted cautiously given the small sample size. Additional markers, including 5hmCyt, 8ocodG, and 5hmdU, showed moderate to large effect size estimates (r = 0.730; *p* = 0.144; T = 1), but these findings were not statistically significant. No effect was observed for 5mdC (r = 0).

Overall, due to the small sample sizes and resulting limited statistical power, none of the analyzed effects reached statistical significance after FDR correction. The observed effect sizes and directional changes should therefore be interpreted cautiously, as preliminary indications of possible biological responses rather than confirmatory evidence. These findings highlight the need for further investigation in larger groups to verify the observed patterns.

Analysis of factor weights indicated that the first principal component (PC1) differentiated strongly between body size measures and changes in epigenetic markers. High positive loadings were observed for 5hmdC ∆ (0.405) and 5mdC ∆ (0.391), whereas negative loadings were associated with weight (−0.385), BMI (−0.310), and height (−0.302).

**Figure 2:**
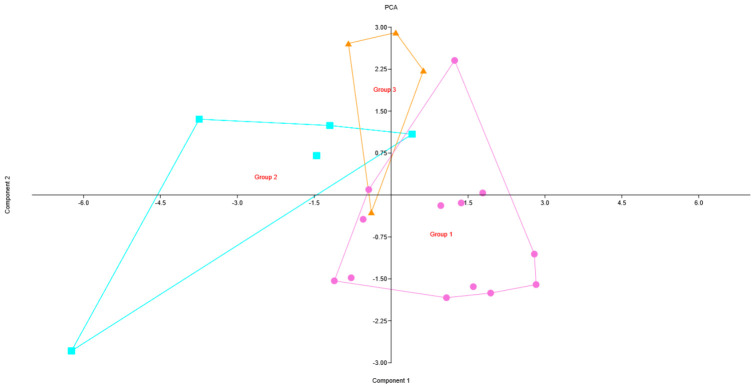
Principal Component Analysis (PCA) plot shows the distribution of samples along the first two principal components. The plot illustrates the separation/clustering of groups based on the analyzed variables.

The second principal component (PC2) reflected the influence of age and lifestyle factors on oxidative modifications. PC2 showed the highest positive loadings for age (0.516), 5hmdU (0.386), 5hmUra (0.381) and smoking (0.334). Given the exploratory nature of this PCA—necessitated by the high variable-to-sample ratio and the inclusion of binary variables—the PC1 versus PC2 graph (Figure 2) suggested a potential, preliminary clustering among the three patient groups (*n* = 13, *n* = 5, *n* = 4) rather than a definitive separation. Furthermore, because Group 3 is characterized by an older demographic and a higher prevalence of smokers, the variations in uracil derivatives (5-hmdU and 5-hmUra) loading on PC2 must be interpreted with caution. These specific modifications may be confounded by age and tobacco use, and therefore cannot be definitively attributed solely to occupational polyurethane (PUR) exposure. The PC1 versus PC2 graph (Figure 2) suggests only a preliminary clustering pattern rather than definitive group separation.

Groups 1 and 2 were almost completely separated along the horizontal axis (PC1). Group 1, defined by positive PC1 values, included participants with lower weight and BMI, and showed a larger increase in 5mdC ∆ and 5hmdC ∆ after exposure. Group 2, in the negative PC1 region, consisted of participants with higher weight and BMI and exhibited a smaller increase in 5mdC and 5hmdC after exposure.

Although some separation between groups was visually apparent, this pattern must be interpreted with caution. Group 3, consisting of four individuals, was characterized by higher age and a greater prevalence of smoking, both of which were strongly associated with PC2 loadings. Therefore, the observed variation in uracil derivatives (5hmdU and 5hmUra) may be confounded by these factors and cannot be attributed solely to occupational exposure. Importantly, as this Principal Component Analysis was performed using only 21 observations while incorporating numerous clinical and biochemical variables, it is highly susceptible to overfitting. The apparent separation between groups must be interpreted with extreme caution, as it may largely reflect the very small sample size rather than true biological clustering. Validation in an independent dataset is necessary before assigning definitive biological significance to these clusters.

## 3. Discussion

The transition from physiological homeostasis to pathological states is a complex, multi-stage process driven by the synergistic interplay of genetic mutations, epigenetic dysregulation, and oxidative damage [1]; study suggests that even a standard workweek in high-exposure industrial sectors may be associated with a measurable shift in the urinary DNA modification landscape. These findings identify 5-hmdUand the epigenetic turnover ratio as potential candidate indicators of genotoxic strain, which warrant further comparative studies against the traditional “gold standard” marker, 8-oxodG.

### 3.1. Pyrimidine Instability and Particulate Matter

The most striking finding of this study is the 221.5% surge in urinary 5-hmdU among workers in the foam processing sector (Group 3). While these workers are exposed to polymer dust and melamine-related compounds, this observation must be interpreted with strict caution given the small sample size and exploratory design of our study. Specifically, because individuals in Group 3 are older and include a higher proportion of smokers, demographic and lifestyle factors act as major confounders. While 8-oxodG has long been utilized to monitor purine oxidation [14], the altered pyrimidine oxidation profile observed in this cohort cannot be definitively attributed to occupational hazards. Further large-scale studies are necessary to disentangle the potential effects of particulate matter exposure from those driven by age and smoking habits.

Inhalation of fine polymer dust is known to trigger an “oxidative burst” in alveolar macrophages and neutrophils, releasing large quantities of reactive oxygen species (ROS). These ROS target nitrogenous bases; however, the accumulation of 5-hmdU, a product of thymine oxidation or 5-hmdC deamination, presents an interesting observation. It might be speculated that this accumulation is linked to a localized saturation of the Base Excision Repair (BER). However, without measuring systemic expression levels or the enzymatic velocity of key glycosylases such as SMUG1 or TDG, this concept must be treated purely as an unverified mechanistical hypothesis. Theoretically, if these repair enzymes are unable to keep pace with the rate of lesion formation, an excess of these mutagenic uracil derivatives would be excreted in urine, reflecting a global “turnover” of DNA damage [18,20]. The high directional consistency of this observation (100% of Group 3) suggests 5-hmdU may serve as a potential, sector-specific biomarker for individuals handling melamine-modified polymers.

### 3.2. Epigenetic Stagnation and Altered Dynamics in the TET Pathway

This pilot study raises the possibility that various industrial stressors could interact with the epigenetic machinery via divergent mechanisms, as suggested by variations in the Epigenetic Turnover Ratio (5-hmdC/5-mdC).

#### 3.2.1. Isocyanates and the Shutdown of Genomic Plasticity

Workers exposed to isocyanates exhibited a synchronized depletion of 5-mdC (−17.2%) and 5-hmdC (−29.1%). This pattern, coupled with a 16.3% drop in the turnover ratio, indicates a state of epigenetic stagnation. Isocyanates are highly reactive electrophiles (R-N=C=O) that can directly interact with the thiol groups (-SH) of essential enzymes.

We hypothesize that isocyanate exposure might lead to the direct inhibition of both DNA DNMTs) and the TET family. Because no experiments evaluating DNMT or TET activity were performed, these mechanistic explanations are presented strictly as hypotheses. The reduction in 5-mdC suggests a compromised ability to maintain the primary methylation sequence [5], while the drop in the turnover ratio indicates a blockade of the active demethylation flux [9]. Such stagnation could reduce the cell’s capacity for epigenetic reprogramming, a prerequisite for maintaining chromosomal stability and responding to environmental stress [38]. This hypothesized “shutdown” of the epigenetic landscape could theoretically serve as an early indicator of genomic instability, a concept that merits longitudinal investigation [39].

#### 3.2.2. Particulate Matter and Epigenetic Turnover

In contrast, the foam processing group manifested a 133.3% increase in the turnover ratio and a 108.7% increase in 5-hmCyt. This reflects a state of “metabolic hyper-activation.” The sharp rise in 5-hmdC relative to 5-mdC suggests that the genome is undergoing rapid, TET-mediated epigenetic remodeling as an adaptive response to particulate-induced inflammation. Given that 5-hmdC is essential for gene expression regulation in transcriptionally active regions [10], this hyper-turnover may represent a systemic attempt to activate stress-response genes and repair pathways.

### 3.3. Individual Susceptibility and Biomarker Variability

The Beauty sector (Group 1) presented a unique challenge in data interpretation due to extreme inter-individual heterogeneity. While median shifts were negligible, the presence of specific “hyper-responders” highlights the role of individual susceptibility. Lash stylists are exposed to formaldehyde and methyl methacrylate (MMA), both recognized as potent epigenetic disruptors [40].

Formaldehyde can induce DNA-protein crosslinks, which physically obstruct the movement of DNMTs and TET enzymes along the chromatin [11]. The high variance in biomarker levels within this group likely reflects differences in workstation ventilation, personal protective equipment usage, and genetic polymorphisms in antioxidant enzymes. Our findings suggest that for the beauty industry, longitudinal monitoring must focus on individual delta shifts rather than cohort-level averages to effectively identify workers at risk of clinical pathology.

### 3.4. Clinical Translation and International Context

The global scale of occupational chemical exposure, affecting over 1 billion workers (ILO/WHO data), requires biomonitoring tools that are both sensitive and non-invasive. Urine has emerged as a superior matrix for this purpose, reflecting the real-time efficiency of systemic repair systems [20].

Our results demonstrate that urinary signatures of 5-hmdC and 5-mdC provide a comprehensive view of epigenetic health. Furthermore because research shows that global hypomethylation and the loss of 5-hmdC may be hallmarks of carcinogenesis [7,8,39], the detection of these shifts within a single workweek might underscore the urgency of re-evaluating occupational safety limits. The “uracil explosion” in Group 3 specifically identifies 5-hmdU as an exploratory marker for sectors handling melamine-modified polymers, where traditional 8-oxodG monitoring may fail to capture the full extent of genotoxic strain. However, the authors recognize that, as this is a pilot study, these conclusions require validation in larger, independent cohorts with long-term follow-up.

### 3.5. Diagnostic and Clinical Significance of Molecular Markers

To provide a broader biological context, the epigenetic and oxidative DNA modifications evaluated in this study are widely recognized for their roles in long-term genomic stability and chronic disease pathogenesis. Table 4 summarizes the current state of the literature regarding these biomarkers in oncology and neurodegenerative research. Importantly, it must be emphasized that the short-term urinary fluctuations observed over a 5-day workweek in this pilot study represent acute, transient biological responses. These temporal shifts cannot be directly extrapolated to chronic disease risks, nor do they imply long-term diagnostic or prognostic outcomes. Table 4 is provided strictly as a conceptual overview of the pathways and clinical contexts these markers generally reflect in the broader literature, completely decoupled from the acute occupational exposures analyzed here.

#### Clinical Translation: From Occupational Stress to Early Detection

The urinary excretion of modified nucleosides reflects the global turnover of DNA damage and the real-time efficiency of systemic repair mechanisms [20]. Recent breakthroughs have demonstrated that urinary signatures of 5-hmdC and 5-mdC provide a comprehensive view of epigenetic health with higher sensitivity than plasma-based assays [21].

Our study bridges the gap between occupational health and molecular oncology by suggesting that these critical signatures shift within a single standard workweek. Specifically, the observed increase in 5-hmdU and the loss of epigenetic turnover efficiency (ratio decrease) identified in industrial cohorts may represent early indicators of genotoxic strain. Pending future validation in adequately powered studies, monitoring these “sixth bases” of the genome could eventually aid in the identification of individuals at risk and the implementation of protective measures prior to the transition to clinical pathology [41,42].

**Table 4:** Summary of Molecular Biomarkers in Clinical Diagnostics and Research.

Marker	Clinical Application	Diagnostic/Prognostic Significance	Key References
5-mdC	Pediatric ALL	Tracks chemotherapy response; levels in urine significantly higher at diagnosis, normalizing post-treatment.	[31]
5-mdC	Colorectal & Breast Cancer	Global hypomethylation as a marker of genomic instability and poor survival outcomes.	[43,44]
5-hmdC	Colorectal Cancer (CRC)	High diagnostic accuracy in liquid biopsy (AUC = 0.95); markers track demethylation during tumor development.	[42,45]
5-hmCyt (5-hmC)	Malignant Melanoma	Complete loss in 100% of metastatic cases; critical for differentiating benign nevi from malignant cells.	[11]
5-hmC	Prostate Cancer (mCRPC)	Predicts resistance to ADT therapy; associated with gene body profiling of *AR*, *PTEN*, and *RB1*.	[46,47]
5-hmC	Non-Small Cell Lung Cancer	Superior prognostic value for overall survival (OS) compared to traditional TNM staging.	[41]
5-hmC	Papillary Thyroid Cancer	Marker of aggressive phenotype and predictor of lymph node metastasis.	[48]
5-hmdU/5-hmUra	Colorectal Cancer	Marker of oxidative DNA damage and active demethylation; elevated urinary levels in patients with CRC compared with healthy individuals.	[18,23]
8-oxodG	Solid Tumors (Meta-analysis)	Strong correlation with short overall survival; established biomarker of systemic oxidative load.	[49]
8-oxodG	Breast Cancer Risk	Elevated levels in peripheral blood leukocyte DNA serve as a risk assessment marker.	[50]
8-oxodG	Neurodegeneration (AD/PD)	Accumulation in mitochondrial and nuclear DNA; linked to alpha-synuclein aggregation in Parkinson’s.	[27,28]
DNA Methylation	Bladder Cancer	Non-invasive diagnosis using urine sediment; focuses on promoter hypermethylation of suppressor genes.	[51,52]

## 4. Materials and Methods

### 4.1. Samples Collection

This study was designed as a prospective, repeated-measures pilot investigation conducted over a single five-day working week to evaluate acute epigenetic responses to occupational chemical exposures. Urine samples were collected at two standardized time points: immediately before the onset of occupational exposure (pre-exposure baseline) and directly following the completion of the working week (post-exposure).

Three occupational cohorts with well-defined and distinct chemical exposure profiles were recruited for biomonitoring: Group 1 comprised professionals from the beauty and cosmetics sector (*n* = 13 individuals); Group 2 consisted of workers with documented isocyanate exposure (*n* = 5 individuals); and Group 3 included personnel engaged in precision foam-cutting operations (*n* = 4 individuals).

In Group 2 (Isocyanates), biological samples were initially collected from 5 participants at both pre- and post-exposure time points. However, for one participant, post-exposure marker concentrations were not detected and could not be quantified; consequently, this pair was excluded from the paired Wilcoxon signed-rank test for the affected markers, yielding N = 4 complete pairs used in the statistical analysis (Table 3). Marker-specific sample sizes reported in Table 3 (e.g., *n* = 10–13 for Group 1) reflect analogous exclusions of incomplete pairs due to non-detectable values. Consequently, a total of 43 biological samples were tested.

The limited cohort size reflects the highly specialized nature of the targeted exposure profiles and the exploratory scope of the study; statistical analyses were therefore performed using non-parametric methods appropriate for small sample sizes, including paired analyses for repeated measures. Participants were classified based solely on their occupational assignment. Actual exposure levels to isocyanates, melamine, polymer dust, or other chemicals were not directly quantified via environmental or biological exposure measurements. Additionally, no specific dietary restrictions were implemented prior to urine collection.

First-morning midstream urine specimens were self-collected by participants into sterile polypropylene containers. To minimize oxidative degradation of target analytes, particularly 8-oxo-7,8-dihydro-2′-deoxyguanosine (8-oxodG), which is susceptible to ex vivo artifactual oxidation, samples were immediately shielded from light, placed on ice, and transported to the laboratory within two hours of collection at 4–5 °C. All specimens were aliquoted and stored at −80 °C until downstream epigenetic analysis.

Because of the longitudinal, self-controlled nature of the study design, where each participant served as their own internal baseline reference, the statistical and analytical focus was directed toward intra-individual biomarker kinetics rather than inter-group sample maximization.

### 4.2. Determination of Epigenetic Modifications and 8-oxodG Levels in Urine

Two-dimensional ultra-performance liquid chromatography with tandem mass spectrometry (2D UPLC-MS/MS) was used for the epigenetic modification analysis of the urine samples (except 5-hmUra). The urine samples were spiked with a mixture of internal standards at a 4:1 volumetric ratio. The 2D UPLC-MS/MS system consists of a gradient pump and autosampler for one-dimensional chromatography, and a gradient pump and tandem quadrupole mass spectrometer with a UNISPRAY ion source were used for two-dimensional chromatography. Both systems were coupled with a column manager equipped with two programmable column heaters and two 2-position 6-port switching valves. The at-column dilution technique was used between the first and second dimensions to improve retention in the trap/transfer column. The sample molecules were then adsorbed to the packing material as very narrow bands that could be eluted with well-resolved, small-volume peaks. A diluting stream of water (0.5 mL/min) was pumped with a Waters 515 isocratic pump and mixed with the first-dimensional column effluent via a UPLC low-dead-volume tee valve. The following columns were used: a CORTECS UPLC T3 column (1.6 µm, 3 mm × 150 mm) with a CORTECS T3 VanGuard precolumn (1.6 µm, 2.1 mm × 5 mm) for the first dimension, a Waters ACQUITY UPLC CSH C18 column (1.7 µm, 2.1 mm × 100 mm) for the second dimension, and a Waters XSelect CSH C18 column (3.5 µm, 3 mm × 20 mm) as the trap/transfer column (all columns supplied by Waters Corporation, Milford, MA, USA). The chromatographic system was operated in heart-cutting mode, which means that selected portions of effluent from the first dimension were loaded onto the trap/transfer column by 6-port valve switching, which served as an “injector” for the second dimension of the chromatography system. Mass spectrometric detection was conducted with a Waters Xevo TQ-S tandem quadrupole mass spectrometer equipped with a UniSpray ionization source (Waters Corporation, Milford, MA, USA). The following common detection parameters were used: source temperature, 150 °C; nitrogen desolvation gas flow, 1000 L/h; nitrogen cone gas flow, 150 L/h; desolvation temperature, 500 °C; and nebulizer gas pressure, 7 bar. Collision-induced dissociation was obtained with argon (6.0 at 3 × 10^−6^ bar pressure) as the collision gas. The instrument response to all the compounds was optimized by the infusion of 10 µM genuine compounds dissolved in water (10 µL/minute) in the mobile phase A stream via the mass spectrometer fluidics system operating in the “mixed” mode via the MassLynx 4.1 software IntelliStart feature.

The quantitative and qualitative transition patterns and the specific settings of the detector are summarized in Table S1 (Supplementary Materials). Among the analyzed compounds by 2D UPLC-MS/MS, only 5-(hydroxymethyl)-2′-deoxyuridine was quantified in negative ionization mode (UniSpray) in a separate, independent analysis. The chromatographic system was operated with MassLynx 4.1 software from Waters. Quantitative analyses were performed via the TargetLynx application. All samples were analyzed with three to six technical replicates. Because 5-hmUra levels fell below the limit of quantification on the 2D UPLC-MS/MS platform, this analyte was quantified using high-performance liquid chromatography for pre-purification followed by gas chromatography with isotope dilution mass spectrometry (LC/GC-MS), a fully validated workflow previously described by Rozalski et al. [23]. The use of stable isotope internal standard in this GC-MS workflow inherently and precisely corrects for analyte recovery and matrix effects. Importantly, because all paired pre- and post-exposure analyses for 5-hmUra were performed exclusively within this internally consistent LC/GC-MS dataset, no cross-platform analytical bias was introduced into the statistical evaluations.

Urinary creatinine was used to correct the analyzed compounds in urine for variability in urine dilution.

### 4.3. Statistical Analysis

Descriptive statistics for variables with non-normal distributions are presented as medians and interquartile ranges (IQR; 25th–75th percentiles). The normality of data distributions was assessed through visual inspection of probability plots and verified using the Shapiro–Wilk test. Given the non-normal distribution of the genetic marker data, non-parametric methods were employed for all subsequent inferential analyses.

To evaluate changes in genetic markers within the same individuals across two time points (pre- and post-occupational exposure), the non-parametric Wilcoxon signed-rank test was utilized, with the sum of ranks (T) serving as the primary test statistic. For groups with high exposure levels characterized by small sample sizes (Isocyanates and PUR processing; N = 4), the Wilcoxon test encounters a formal mathematical limitation that prevents the calculation of a two-tailed raw *p*-value below 0.068. Since this threshold precludes the conventional rejection of the null hypothesis at α = 0.05, statistical inference for these cohorts focused on standardized effect sizes. The effect coefficient (r) was calculated by dividing the absolute value of the standardized Z-statistic by the square root of the number of studied pairs. These r values were interpreted according to Cohen’s criteria, where r < 0.10 indicates a negligible effect 0.10 ≤ r < 0.30 a small effect, 0.30 ≤ r < 0.50 a medium effect, 0.50 ≤ r < 0.70 a large effect, and r ≥ 0.70 a very large effect.

To mitigate the risk of Type I errors arising from multiple hypothesis testing, raw *p*-values were adjusted using the Benjamini–Hochberg procedure to control the False Discovery Rate (FDR). Adjustments were applied independently within each of the three occupational cohorts (Beauty, Isocyanates, and PUR processing), as each represented a distinct chemical exposure profile. The analytical focus remained on intra-group marker alterations rather than direct inter-group comparisons.

Principal Component Analysis (PCA) was performed to reduce data dimensionality and visualize biological variation. The first two principal components (PC1 and PC2) were retained based on the screen plot criterion (broken-stick model), which confirmed that both components captured genuine, non-random biological variance, while subsequent components primarily represented stochastic noise. PC1 and PC2 accounted for 25.52% and 16.04% of the total variance, respectively, cumulatively explaining 41.56% of the dataset’s variance. PCA as a complementary method to statistical analysis.

Principal Components Analysis (PCA) was conducted to systematically assess the effects of exposure and identify similarities among patients. A correlation matrix (Correlation PCA) was applied to standardize the data and remove the influence of varying measurement units. To reduce individual background effects and emphasize biological changes, changes (∆) for each of the seven health measures were calculated using the following formula:

∆ = Value AFTER − Value BEFORE

Continuous and binary variables from the biomedical survey were included. These comprised age, height, weight, BMI, cigarette smoking status, Euthyrox intake, vitamin C supplementation, and selected family burdens (diabetes, thyroid disease, cancer). The data matrix contained 21 observations (patients) and 17 variables. Component significance was evaluated using a scree plot and compared with the broken-stick model. Group structure was visualized as a scatter plot of objects with convex hulls. All calculations were performed in PAST software (version 4.16).

All statistical procedures and visualizations were conducted using Statistica software (version 13). Except where mathematically constrained by sample size, the threshold for statistical significance was set at *p* < 0.05.

## 5. Conclusions

This study reveals rapid shifts in the urinary DNA modification landscape among different occupational groups within a single standard workweek. These exploratory findings suggest two distinct molecular signatures of genotoxic strain, specifically oxidative pyrimidine instability and epigenetic stagnation. While the apparent accumulation of urinary 5-hmdU identified in the foam-cutting sector is a notable finding, its direct attribution to particulate exposure remains inconclusive due to the concurrent confounding effects of age and smoking in this cohort. Conversely, the synchronized depletion of 5-mdC and 5-hmdC in the isocyanate sector might indicate a disruption of genomic methylation maintenance. The trends observed in these epigenetic and oxidative markers, precisely quantified via 2D UPLC-MS/MS, point toward their potential utility as non-invasive early indicators. Subject to further validation in larger, well-matched cohorts to account for demographic and lifestyle factors, these molecular signatures might eventually provide a window for early monitoring of systemic biological strain prior to the onset of clinical pathology. Incorporating the evaluation of these “sixth bases” of the genome into occupational health research could eventually contribute to the precision of risk assessment and long-term disease prevention strategies.

### Limitation of the Study

Several aspects of this exploratory study design should be contextualized. First, the specialized industrial cohorts (Groups 2 and 3) were framed as highly targeted, screening-level sub-groups. This focused approach was strictly dictated by the exceptional specialization and narrow accessibility of these distinct occupational sectors. This resulted in an extremely small sample size that substantially limits statistical power and the reliability of broad conclusions.

Second, as is common in field-based biomonitoring, urinary biomarker concentrations can be subject to variability from factors such as hydration status, renal function, diet, circadian rhythms, and medications. While creatinine normalization was applied to mitigate these effects, the potential influence of such variables cannot be entirely ruled out.

Third, the exposure matrices in this study were defined nominally by employment sector rather than by actual personal passive samplers or stationary industrial monitoring data. The lack of precise airborne toxicant concentrations (e.g., mg/m^3^ of chemical agents or particulate dust counts) makes it impossible to establish definitive dose–response relationships. While our approach reflects real-world occupational scenarios, incorporating precise chemical measurements in future research is essential to elucidate the direct causal relationships between specific agents and the observed biomarker shifts.

Finally, the molecular mechanisms discussed in this exploratory study—such as potential DNMT/TET modulation, BER pathway dynamics, or ROS generation—are presented as theoretical frameworks. As our study was primarily designed to assess non-invasive biomarker kinetics, these mechanistic hypotheses provide a valuable direction for future targeted experimental validation.

## Figures and Tables

**Table 1:** Median percentage change (%Δ) in urinary DNA modifications following a standard workweek.

Marker	Group 1 (Beauty)	Group 2 (Isocyanates)	Group 3 (PUR Processing)
5-mdC	+0.7%	−17.2%	+3.75%
5-hmCyt	−23.2%	−22.8%	+108.7%
5-hmdC	+8.5%	−29.1%	+14.1%
8-oxodG	+4.9%	−9.1%	−14.9%
5-hmdU	+13.9%	+103.3%	+221.5%
5-hmUra	−21.8%	+11.5%	+46.84%

**Table 2:** Workweek-induced changes in the Epigenetic Turnover Ratio.

Study Group	Pre-Exposure (Median)	Post-Exposure (Median)	Δ Ratio (%)	Observed Trends
Group 1 (Beauty)	0.92	0.98	+6.5%	Homeostatic Stability
Group 2 (Isocyanates)	0.43	0.36	−16.3%	TET Pathway Inhibition
Group 3 (PUR processing)	0.12	0.28	+133.3%	Metabolic Hyper-activation

**Table 3:** Statistical significance (*p*-values) of urinary DNA modification changes during a standard workweek (Wilcoxon matched-pairs signed-rank test), effect size and False Discovery Rate.

Groups	Parameters	Wilcoxon Paired Order Test						Effect Size *		FDR Correction **	
		Median Before ***	Median After ***	Statistics T	Statistics Z	*p*-Value	N ****	r	Interpretation	Rank	*p*_FDR
Group 1 (Beauty)	5mdC	0.51 (0.43–0.59)	0.52 (0.33–0.97)	37	0.16	0.875	12	0.045	No effect	6	0.969
	5hmCyt	1.7 (1.31–4.92)	1.3 (1.09–2.07)	14	1.38	0.169	10	0.435	Medium	2	0.969
	5hmdC	0.47 (0.41–0.59)	0.51 (0.31–0.7)	39	0.04	0.969	12	0.011	No effect	7	0.969
	8ocodG	0.66 (0.59–0.86)	0.69 (0.46–0.89)	35	0.73	0.463	13	0.204	Small	3	0.969
	5hmdU	0.9 (0.62–5.94)	1.02 (0.35–2.19)	26	0.62	0.534	11	0.188	Small	4	0.969
	5hmUra	9.02 (6.85–11.53)	7.06 (6.7–11.82)	39	0.45	0.650	13	0.126	Small	5	0.969
Group 2 (Isocyanates)	5mdC	2.21 (2.16–2.29)	1.83 (1.82–1.88)	0	1.83	0.068	4	0.913	Very large	1	0.342
	5hmCyt	1.77 (1.12–2.05)	1.37 (1.12–1.83)	4	0.37	0.715	4	0.183	Small	6	0.715
	5hmdC	0.95 (0.89–1.16)	0.67 (0.60–0.72)	2	1.10	0.273	4	0.548	Large	2	0.342
	8ocodG	0.91 (0.58–1.4)	0.83 (0.68–1.26)	2	1.10	0.273	4	0.548	Large	3	0.342
	5hmdU	0.715 (0.705–0.91)	1.46 (0.56–2.62)	1	1.07	0.285	4	0.535	Large	5	0.342
	5hmUra	3.92 (3.73–4.08)	4.37 (3.24–5.83)	2	1.10	0.273	4	0.548	Large	4	0.342
Group 3 (PUR processing)	5mdC	1.015 (0.82–1.31)	1.05 (0.93–1.08)	5	0	1.000	4	0	No effect	6	1.0
	5hmCyt	0.87 (0.82–1.12)	1.815 (1.63–1.84)	1	1.46	0.144	4	0.730	Very large	2	0.216
	5hmdC	0.21 (0.1–0.36)	0.24 (0.18–0.33)	4	0.37	0.715	4	0.183	Small	5	0.856
	8ocodG	0.80 (0.71–1.0)	0.68 (0.64–0.83)	1	1.46	0.144	4	0.730	Very large	3	0.216
	5hmdU	1.07 (0.78–1.43)	3.43 (2.46–4.43)	1	1.46	0.144	4	0.730	Very large	4	0.216
	5hmUra	3.43 (2.77–4.0)	5.04 (4.21–5.43)	0	1.83	0.068	4	0.913	Very large	1	0.216

* The magnitude of the effect was estimated using Rosenthal’s r-factor, and the obtained values were interpreted according to the classical thresholds proposed by Cohen (0.1—weak effect; 0.3—moderate effect; 0.5—strong effect). ** FDR CORRECTION (Benjamini–Hochberg). *** Median (25–75%). **** N reflects the number of complete pre–post pairs available for each marker after exclusion of samples with not detected values in either time point; incomplete pairs were not included in the Wilcoxon signed-rank test.

## Data Availability

The original contributions presented in this study are included in the article/Supplementary Materials. Further inquiries can be directed to the corresponding authors.

## Supplementary Materials

The following supporting information can be downloaded at: https://www.mdpi.com/article/10.3390/ijms27146411/s1.

## References

[1] Vasanthakumar A., Godley L.A. (2015). 5-Hydroxymethylcytosine in Cancer: Significance in Diagnosis and Therapy. Cancer Genet..

[2] Fleming A.M., Ding Y., Burrows C.J. (2017). Oxidative DNA Damage Is Epigenetic by Regulating Gene Transcription via Base Excision Repair. Proc. Natl. Acad. Sci. USA.

[3] Deaton A.M., Bird A. (2011). CpG Islands and the Regulation of Transcription. Genes Dev..

[4] Reik W. (2007). Stability and Flexibility of Epigenetic Gene Regulation in Mammalian Development. Nature.

[5] Jones P.A., Baylin S.B. (2002). The Fundamental Role of Epigenetic Events in Cancer. Nat. Rev. Genet..

[6] Bird A. (2002). DNA Methylation Patterns and Epigenetic Memory. Genes Dev..

[7] Jeschke J., Collignon E., Fuks F. (2016). Portraits of TET-Mediated DNA Hydroxymethylation in Cancer. Curr. Opin. Genet. Dev..

[8] Rasmussen K.D., Helin K. (2016). Role of TET Enzymes in DNA Methylation, Development, and Cancer. Genes Dev..

[9] Tahiliani M., Koh K.P., Shen Y., Pastor W.A., Bandukwala H., Brudno Y., Agarwal S., Iyer L.M., Liu D.R., Aravind L. (2009). Conversion of 5-Methylcytosine to 5-Hydroxymethylcytosine in Mammalian DNA by MLL Partner TET1. Science.

[10] Kriaucionis S., Heintz N. (2009). The Nuclear DNA Base 5-Hydroxymethylcytosine Is Present in Purkinje Neurons and the Brain. Science.

[11] Lian C.G., Xu Y., Ceol C., Wu F., Larson A., Dresser K., Xu W., Tan L., Hu Y., Zhan Q. (2012). Loss of 5-Hydroxymethylcytosine Is an Epigenetic Hallmark of Melanoma. Cell.

[12] Ehrlich M. (2009). Dna Hypomethylation In Cancer Cells. Epigenomics.

[13] Pfeifer G.P., Kadam S., Jin S.-G. (2013). 5-Hydroxymethylcytosine and Its Potential Roles in Development and Cancer. Epigenet. Chromatin.

[14] Kumar K., Fornace A.J., Suman S. (2024). 8-OxodG: A Potential Biomarker for Chronic Oxidative Stress Induced by High-LET Radiation. DNA.

[15] Pfaffeneder T., Spada F., Wagner M., Brandmayr C., Laube S.K., Eisen D., Truss M., Steinbacher J., Hackner B., Kotljarova O. (2014). Tet Oxidizes Thymine to 5-Hydroxymethyluracil in Mouse Embryonic Stem Cell DNA. Nat. Chem. Biol..

[16] Olinski R., Starczak M., Gackowski D. (2016). Enigmatic 5-Hydroxymethyluracil: Oxidatively Modified Base, Epigenetic Mark or Both?. Mutat. Res./Rev. Mutat. Res..

[17] Rusmintratip V. (2000). Examination of the DNA Substrate Selectivity of DNA Cytosine Methyltransferases Using Mass Tagging. Nucleic Acids Res..

[18] Skalska-Bugala A., Siomek-Gorecka A., Banaszkiewicz Z., Olinski R., Rozalski R. (2022). Urinary Measurement of Epigenetic DNA Modifications and 8-oxodG as Possible Noninvasive Markers of Colon Cancer Evolution. Int. J. Mol. Sci..

[19] Shilnikova N., Momoli F., Karyakina N., Krewski D. (2025). Review of Non–Invasive Biomarkers as a Tool for Exposure Characterization in Human Health Risk Assessments. J. Toxicol. Environ. Health Part B.

[20] Roszkowski K., Olinski R. (2012). Urinary 8-Oxoguanine as a Predictor of Survival in Patients Undergoing Radiotherapy. Cancer Epidemiol. Biomark. Prev..

[21] Xie W., Li X., Chen H., Chu J., Zhang L., Tang B., Huang W., Li L., Lin J., Dong Y. (2024). 5-Hydroxymethylcytosine Profiles of cfDNA in Urine as Diagnostic, Differential Diagnosis and Prognostic Markers for Multiple Myeloma. Cancer Med..

[22] Guo C., Li X., Wang R., Yu J., Ye M., Mao L., Zhang S., Zheng S. (2016). Association between Oxidative DNA Damage and Risk of Colorectal Cancer: Sensitive Determination of Urinary 8-Hydroxy-2′-Deoxyguanosine by UPLC-MS/MS Analysis. Sci. Rep..

[23] Rozalski R., Gackowski D., Siomek-Gorecka A., Starczak M., Modrzejewska M., Banaszkiewicz Z., Olinski R. (2015). Urinary 5-Hydroxymethyluracil and 8-Oxo-7,8-Dihydroguanine as Potential Biomarkers in Patients with Colorectal Cancer. Biomarkers.

[24] Mao L., Guo C., Zheng S. (2018). Elevated Urinary 8-Oxo-7,8-Dihydro-2′-Deoxyguanosine and Serum Uric Acid Are Associated with Progression and Are Prognostic Factors of Colorectal Cancer. OTT.

[25] Loft S., Svoboda P., Kasai H., Tjønneland A., Vogel U., Møller P., Overvad K., Raaschou-Nielsen O. (2006). Prospective Study of 8-Oxo-7,8-Dihydro-2′-Deoxyguanosine Excretion and the Risk of Lung Cancer. Carcinogenesis.

[26] Cooke M.S., Olinski R., Evans M.D. (2006). Does Measurement of Oxidative Damage to DNA Have Clinical Significance?. Clin. Chim. Acta.

[27] Gackowski D., Rozalski R., Siomek A., Dziaman T., Nicpon K., Klimarczyk M., Araszkiewicz A., Olinski R. (2008). Oxidative Stress and Oxidative DNA Damage Is Characteristic for Mixed Alzheimer Disease/Vascular Dementia. J. Neurol. Sci..

[28] Basu S., Je G., Kim Y.-S. (2015). Transcriptional Mutagenesis by 8-oxodG in α-Synuclein Aggregation and the Pathogenesis of Parkinson’s Disease. Exp. Mol. Med..

[29] Chiorcea-Paquim A.-M. (2022). 8-Oxoguanine and 8-Oxodeoxyguanosine Biomarkers of Oxidative DNA Damage: A Review on HPLC–ECD Determination. Molecules.

[30] Hu C.-W., Liu H.-H., Li Y.-J., Chao M.-R. (2012). Direct Analysis of 5-Methylcytosine and 5-Methyl-2′-Deoxycytidine in Human Urine by Isotope Dilution LC-MS/MS: Correlations with N-Methylated Purines and Oxidized DNA Lesions. Chem. Res. Toxicol..

[31] Rozalski R., Gackowski D., Skalska-Bugala A., Starczak M., Siomek-Gorecka A., Zarakowska E., Modrzejewska M., Dziaman T., Szpila A., Linowiecka K. (2021). The Urinary Excretion of Epigenetically Modified DNA as a Marker of Pediatric ALL Status and Chemotherapy Response. Sci. Rep..

[32] Cooke M.S., Barregard L., Mistry V., Potdar N., Rozalski R., Gackowski D., Siomek A., Foksinski M., Svoboda P., Kasai H. (2009). Interlaboratory Comparison of Methodologies for the Measurement of Urinary 8-Oxo-7,8-Dihydro-2′-Deoxyguanosine. Biomarkers.

[33] Chen G.G., Gross J.A., Lutz P.-E., Vaillancourt K., Maussion G., Bramoulle A., Théroux J.-F., Gardini E.S., Ehlert U., Bourret G. (2017). Medium Throughput Bisulfite Sequencing for Accurate Detection of 5-Methylcytosine and 5-Hydroxymethylcytosine. BMC Genom..

[34] Liu Y., Siejka-Zielińska P., Velikova G., Bi Y., Yuan F., Tomkova M., Bai C., Chen L., Schuster-Böckler B., Song C.-X. (2019). Bisulfite-Free Direct Detection of 5-Methylcytosine and 5-Hydroxymethylcytosine at Base Resolution. Nat. Biotechnol..

[35] Halliwell D.O., Honig F., Bagby S., Roy S., Murrell A. (2025). Double and Single Stranded Detection of 5-Methylcytosine and 5-Hydroxymethylcytosine with Nanopore Sequencing. Commun. Biol..

[36] (2021). Exposure to Hazardous Chemicals at Work and Resulting Health Impacts: A Global Review.

[37] (2026). Dangerous Substances.

[38] Putiri E.L., Robertson K.D. (2011). Epigenetic Mechanisms and Genome Stability. Clin. Epigenet..

[39] Tian Y., Lin A., Gan M., Wang H., Yu D., Lai C., Zhang D., Zhu Y., Lai M. (2017). Global Changes of 5-Hydroxymethylcytosine and 5-Methylcytosine from Normal to Tumor Tissues Are Associated with Carcinogenesis and Prognosis in Colorectal Cancer. J. Zhejiang Univ. Sci. B.

[40] Sobral A.F., Cunha A., Costa I., Silva-Carvalho M., Silva R., Barbosa D.J. (2025). Environmental Xenobiotics and Epigenetic Modifications: Implications for Human Health and Disease. J. Xenobiot..

[41] Shao J., Olsen R.J., Kasparian S., He C., Bernicker E.H., Li Z. (2024). Cell-Free DNA 5-Hydroxymethylcytosine Signatures for Lung Cancer Prognosis. Cells.

[42] Puddu F., Johansson A., Modat A., Scotcher J., Sethi R., Yu S., Harding N., Hill M., Lleshi E., Lumby C. (2026). 5-Methylcytosine and 5-Hydroxymethylcytosine Are Synergistic Biomarkers for Early Detection of Colorectal Cancer. Commun. Med..

[43] Feinberg A.P., Vogelstein B. (1983). Hypomethylation Distinguishes Genes of Some Human Cancers from Their Normal Counterparts. Nature.

[44] Linowiecka K., Guz J., Dziaman T., Urbanowska–Domańska O., Zarakowska E., Szpila A., Szpotan J., Skalska-Bugała A., Mijewski P., Siomek-Górecka A. (2024). The Level of Active DNA Demethylation Compounds in Leukocytes and Urine Samples as Potential Epigenetic Biomarkers in Breast Cancer Patients. Sci. Rep..

[45] Chung D.C., Gray D.M., Singh H., Issaka R.B., Raymond V.M., Eagle C., Hu S., Chudova D.I., Talasaz A., Greenson J.K. (2024). A Cell-Free DNA Blood-Based Test for Colorectal Cancer Screening. N. Engl. J. Med..

[46] Sjöström M., Zhao S.G., Levy S., Zhang M., Ning Y., Shrestha R., Lundberg A., Herberts C., Foye A., Aggarwal R. (2022). The 5-Hydroxymethylcytosine Landscape of Prostate Cancer. Cancer Res..

[47] Li Q., Huang C.-C., Huang S., Tian Y., Huang J., Bitaraf A., Dong X., Nevalainen M.T., Patel M., Wong J. (2025). 5-Hydroxymethylcytosine Sequencing of Plasma Cell-Free DNA Identifies Epigenomic Features in Prostate Cancer Patients Receiving Androgen Deprivation Therapies. Commun. Med..

[48] Tong M., Gao S., Qi W., Shi C., Qiu M., Yang F., Bai S., Li H., Wang Z., Sun Z. (2019). 5-Hydroxymethylcytosine as a Potential Epigenetic Biomarker in Papillary Thyroid Carcinoma. Oncol. Lett..

[49] Qing X., Shi D., Lv X., Wang B., Chen S., Shao Z. (2019). Prognostic Significance of 8-Hydroxy-2′-Deoxyguanosine in Solid Tumors: A Meta-Analysis. BMC Cancer.

[50] Dziaman T., Huzarski T., Gackowski D., Rozalski R., Siomek A., Szpila A., Guz J., Lubinski J., Olinski R. (2009). Elevated Level of 8-oxo-7,8-dihydro-2′-deoxyguanosine in Leukocytes of *BRCA1* Mutation Carriers Compared to Healthy Controls. Int. J. Cancer.

[51] Chung W., Bondaruk J., Jelinek J., Lotan Y., Liang S., Czerniak B., Issa J.-P.J. (2011). Detection of Bladder Cancer Using Novel DNA Methylation Biomarkers in Urine Sediments. Cancer Epidemiol. Biomark. Prev..

[52] Beijert I.J., Wever B.M.M., Hentschel A.E., Van Den Burgt Y., Kauer P.C., Lissenberg-Witte B.I., Van Moorselaar R.J.A., Steenbergen R.D.M., Nieuwenhuijzen J.A. (2024). Bladder Cancer Detection in Urine by Novel Methylation Markers. Sci. Rep..

