# Supplementary material for: Urinary DNA Methylation and Hydroxymethylation Dynamics as Candidate Biomarkers of Occupational Chemical Exposure—An Exploratory Pilot Study
Source: Int J Mol Sci. 2026 Jul 19;27(14):6411. doi: 10.3390/ijms27146411 (PMC13411247; doi:10.3390/ijms27146411)
Supplement: Supplementary file 1 [file ijms-27-06411-s001.zip › ijms-4393026-supplementary.pdf]

Table S1. Transition patterns and specific detector settings for all compounds analyzed by 2D UPLC–MS/MS in urine.

compound name		ionization mode	nominal molecular mass (Da)	pseudo-molecular ion formulation	nominal parent ion (Da)	nominal daughter ion	capillary (kV)	cone (V)	collision (eV)
**5-hydroxymethylcytosine**	quantifier	UniSpray+	141	$[M+H]^+$	142	81	1.3	40	18
	qualifier	UniSpray+	141	$[M+H]^+$	142	124	1.3	40	12
**[D_3]-5-hydroxymethylcytosine**	quantifier	UniSpray+	144	$[(M+3)+H]^+$	145	84	1.3	40	18
	qualifier	UniSpray+	144	$[(M+3)+H]^+$	145	127	1.3	40	12
**5-hydroxymethyl-2'-deoxycytidine**	quantifier	UniSpray+	257	$[M+H]^+$	258	124	1.3	35	22
	qualifier	UniSpray+	257	$[M+H]^+$	258	142	1.3	35	10
**[D_3]-5-hydroxymethyl-2'-deoxycytidine**	quantifier	UniSpray+	260	$[(M+3)+H]^+$	261	127	1.3	35	22
	qualifier	UniSpray+	260	$[(M+3)+H]^+$	261	145	1.3	35	10
**8-oxo-2’-deoxyguanosine**	quantifier	UniSpray+	283	$[M+H]^+$	284	168	1.3	30	18
	qualifier	UniSpray+	283	$[M+H]^+$	284	140	1.3	30	18
**[$^{15}N_5$]-8-oxo-2'-deoxyguanosine**	quantifier	UniSpray+	288	$[(M+5)+H]^+$	289	173	1.3	20	15
	qualifier	UniSpray+	288	$[(M+5)+H]^+$	289	145	1.3	20	15
**5 (hydroxymethyl)-2’-deoxyuridine**	quantifier	UniSpray-	258	$[M-H]^-$	257	124	1.3	50	13
	qualifier	UniSpray-	258	$[M-H]^-$	257	214	1.3	50	11
**[$^{13}C_{10}$, $^{15}N_2$]-5 (hydroxymethyl)-2’-deoxyuridine**	quantifier	UniSpray-	270	$[(M+12)-H]^-$	269	131	1.3	50	13
	qualifier	UniSpray-	270	$[(M+12)-H]^-$	269	224	1.3	50	11
**5-methyl-2’-deoxycytidine**	quantifier	UniSpray+	241	$[M+H]^+$	242	126	1.3	30	12
	qualifier	UniSpray+	241	$[M+H]^+$	242	224	1.3	30	12
**[$^{13}C_{10}$, $^{15}N_2$]-5-methyl2’-deoxycytidine**	quantifier	UniSpray+	253	$[(M+12)+H]^+$	254	133	1.3	30	12
	qualifier	UniSpray+	253	$[(M+12)+H]^+$	254	236	1.3	30	12
